# Role of Trabecular Microarchitecture and Its Heterogeneity Parameters in the Mechanical Behavior of Ex Vivo Human L_3_ Vertebrae

**DOI:** 10.1002/jbmr.164

**Published:** 2010-06-18

**Authors:** Julien Wegrzyn, Jean-Paul Roux, Monique E Arlot, Stéphanie Boutroy, Nicolas Vilayphiou, Olivier Guyen, Pierre D Delmas, Roland Chapurlat, Mary L Bouxsein

**Affiliations:** 1 INSERM Research Unit 831, Université de Lyon Lyon, France; 2 Department of Orthopedic Surgery, Pavillon T, Hôpital Edouard Herriot Lyon, France; 3 Orthopedic Biomechanics Laboratory, Beth Israel Deaconess Medical Center and Harvard Medical School Boston, MA, USA

**Keywords:** osteoporosis, vertebra, bone biomechanics, trabecular microarchitecture, heterogeneity

## Abstract

Low bone mineral density (BMD) is a strong risk factor for vertebral fracture risk in osteoporosis. However, many fractures occur in people with moderately decreased or normal BMD. Our aim was to assess the contributions of trabecular microarchitecture and its heterogeneity to the mechanical behavior of human lumbar vertebrae. Twenty-one human L_3_ vertebrae were analyzed for BMD by dual-energy X-ray absorptiometry (DXA) and microarchitecture by high-resolution peripheral quantitative computed tomography (HR-pQCT) and then tested in axial compression. Microarchitecture heterogeneity was assessed using two vertically oriented virtual biopsies—one anterior (Ant) and one posterior (Post)—each divided into three zones (superior, middle, and inferior) and using the whole vertebral trabecular volume for the intraindividual distribution of trabecular separation (Tb.Sp*SD). Heterogeneity parameters were defined as (1) ratios of anterior to posterior microarchitectural parameters and (2) the coefficient of variation of microarchitectural parameters from the superior, middle, and inferior zones. BMD alone explained up to 44% of the variability in vertebral mechanical behavior, bone volume fraction (BV/TV) up to 53%, and trabecular architecture up to 66%. Importantly, bone mass (BMD or BV/TV) in combination with microarchitecture and its heterogeneity improved the prediction of vertebral mechanical behavior, together explaining up to 86% of the variability in vertebral failure load. In conclusion, our data indicate that regional variation of microarchitecture assessment expressed by heterogeneity parameters may enhance prediction of vertebral fracture risk. © 2010 American Society for Bone and Mineral Research.

## Introduction

The risk of osteoporotic fracture is greater at skeletal sites where trabecular bone is predominant (ie, femoral neck, vertebrae, and distal radius). Current diagnostic methods for osteoporosis focus on measurement of bone mineral density (BMD) using dual-energy X-ray absorptiometry (DXA). Although low BMD is among the strongest predictors of fracture risk, it is only one aspect of bone strength, and its predictive value is correspondingly limited because many fractures occur in people with normal BMD.(1) Similarly, in patients receiving antiresorptive treatment, the 5% to 8% improvement in spine BMD does not fully explain the observed 50% to 60% decrease in vertebral fracture incidence.(2) These observations highlight the limitations of BMD as a predictor of fracture risk and the need to also consider other parameters, such as microarchitecture, to improve assessment of skeletal fragility.

Previous in vitro studies have demonstrated that the addition of trabecular microarchitecture to BMD improves the prediction of both trabecular bone mechanical behavior and vertebral strength.(3–7) Moreover, using either histomorphometric methods or peripheral quantitative computed tomography (pQCT) or high-resolution peripheral quantitative computed tomography (HR-pQCT), previous studies have assessed the spatial variation of trabecular microarchitecture in vertebral bodies and shown that the structurally weak regions are located in the superior and anterior regions of the vertebral body.(8–11) Correlations between vertebral strength and trabecular microarchitecture parameters vary among vertebral regions, suggesting that it may be helpful to account for regional variations in trabecular microarchitecture when predicting vertebral fragility.(12) However, despite the potential of trabecular microarchitecture heterogeneity measurements to improve fracture risk assessment, there is limited information about reliable measures of trabecular bone heterogeneity and their clinical utility. Several clinical studies have shown that assessment of the intraindividual distribution of trabecular separation (Tb.Sp*SD) at the peripheral skeletal sites by HR-pQCT or MRI is useful for discrimination of previously fractured versus nonfractured controls,(13–17) but alternate parameters of heterogeneity have not been studied, nor have measurements of Tb.Sp*SD been performed directly on whole vertebrae.

Thus the aim of this study was to assess the contribution of trabecular microarchitecture and its regional variation assessment expressed by heterogeneity parameters to the mechanical behavior of human lumbar vertebrae.

## Materials and Methods

### Bone specimens

Lumbar vertebrae (L_3_) were harvested fresh from 21 lumbar spines of human donors, including 11 men and 10 women, aged 54 to 93 years of age (75 ± 10 years for men and 76 ± 10 years for women). The absence of prevalent fractures or significant bone diseases (ie, bone metastasis, Paget disease, or major osteoarthritis) involving the lumbar spine was confirmed by high-resolution lateral radiographs of the lumbar spine (Faxitron X-Ray Corporation, Lincolnshire, IL, USA). Lumbar osteoarthritis (OA) was evaluated on lateral radiographs according to the Kellgren-Lawrence (K-L) grading scale.(18) Severity of OA was assessed according to the presence of osteophytes and disk narrowing using a four-point scale: normal, minimal, moderate, or severe. Vertebrae with severe OA (grade 4) were excluded. Of those included in the study, 11 (52%), 8 (38%), and 2 (10%) were graded normal, minimal, and moderate OA, respectively.

Areal bone mineral density (aBMD, g/cm^2^) of the vertebral body was measured using DXA (Delphi W, Hologic, Waltham, MA, USA). Bone specimens were maintained frozen at −20°C wrapped in saline-soaked gauze until mechanical testing.(19,20)

### Trabecular microarchitecture and its heterogeneity assessment

**Fig. 1:**
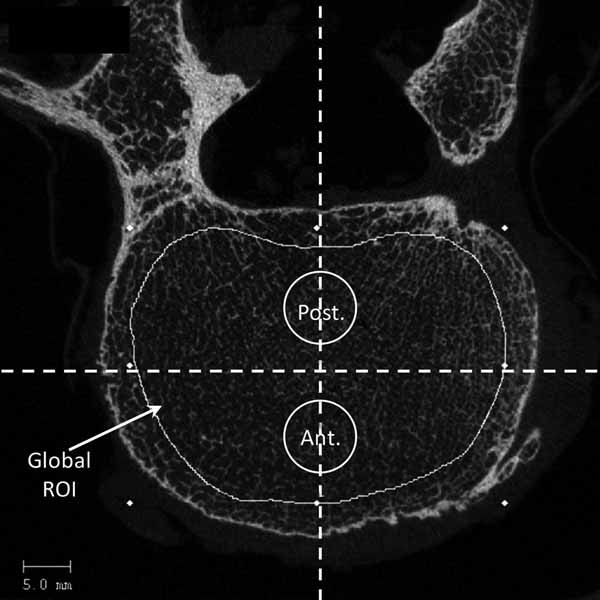
HR-pQCT slice of L_3_ vertebra. Trabecular region of interest (ROI) was defined manually in order to exclude cortical component of the vertebral body. Virtual biopsies were positioned using two lines drawn on the vertebral body, one line for the middle anteroposterior axis and one line for the middle mediolateral axis. Each line divided the vertebral body into four quadrants. Biopsies were strictly centered on the middle anteroposterior axis and on both sides of the mediolateral axis to avoid the cortical shell anteriorly and the venus plexus posteriorly by projection in the vertical direction in the HR-pQCT slice stack.

Image acquisition of the whole frozen vertebral body was performed using HR-pQCT (XtremeCT, Scanco Medical, Bassersdorf, Switzerland). A nominal isotropic voxel size of 82 µm was used (1536 × 1536 pixels; X-ray source: 60 kV, 900 µA). CT slices were perpendicular to the vertebral superoinferior axis. The trabecular region of interest was defined manually in order to exclude cortical component of the vertebral body (Fig. 1).

Bone was segmented using a fixed threshold (175 mg of hydroxyapatite/cm^3^), and 3D trabecular microarchitectural parameters on the whole vertebral body were assessed with software developed for ex vivo analysis (Scanco Medical): bone volume fraction (BV/TV, %), trabecular thickness (Tb.Th*, µm), trabecular separation (Tb.Sp*, µm), trabecular number (Tb.N*, number/mm), degree of anisotropy (DA, number), and structural model index (SMI, number). BV/TV measurement was based on counting voxels. Microarchitecture measurements, which were computed using direct methods (ie, distance-transformation algorithms that do not rely on assumptions about the underlying structure), were designated with an asterisk (eg, Tb.Th*, Tb.Sp*, and Tb.N*).(21,22) DA is defined as the ratio of minimal eigenvalue to maximal eigenvalue and corresponds to a measure of preferential alignment of the trabeculae along a directional axis (1 = isotropic; >1 = anisotropic). SMI is calculated by means of 3D image analysis based on a differential analysis of the triangulated bone surface and reflects the rodlike versus platelike nature of the structure.(23) For ideal plate and rod structures, the SMI values are 0 and 3, respectively.

**Fig. 2:**
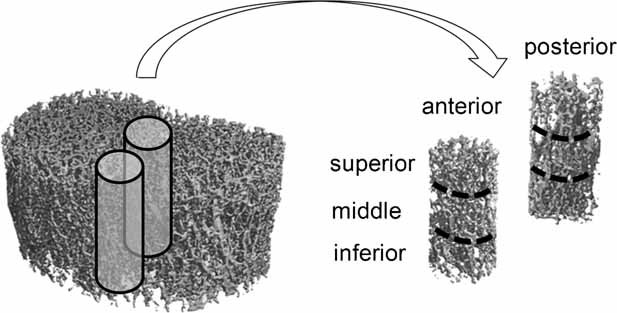
Whole trabecular volume of L_3_ vertebra and the two virtual biopsies (82-µm isotropic voxel size) each divided into three vertical zones (superior, middle, and inferior).

To assess the heterogeneity of vertebral trabecular microarchitecture, microarchitecture parameters were computed for two 8.2-mm-diameter vertically oriented virtual biopsies—one located in the anterior and one in the posterior region, both located along the midline. To position these virtual cores, two lines were defined on the vertebral body—one line for the middle anteroposterior axis and one line for the middle mediolateral axis. Each line divided the vertebral body in four quadrants. Biopsies were strictly centered on the middle anteroposterior axis and just anterior and posterior to the mediolateral axis to avoid the cortical shell anteriorly and the venus plexus posteriorly (Fig. 1). Then each core was divided into three vertical zones (superior, middle, and inferior; Fig. 2).

The following parameters of heterogeneity were computed: (1) *anteroposterior heterogeneity*, the ratio of anteroposterior trabecular microarchitectural parameters (posterior parameter divided by the anterior one: BV/TV_ratio_, SMI_ratio_, Tb.Sp*_ratio_, Tb.Th*_ratio_, Tb.N*_ratio_, and DA_ratio_), (2) *vertical heterogeneity*, the coefficient of variation (standard deviation/mean) of the vertical three zones' trabecular microarchitectural parameters (BV/TV_CV_, SMI_CV_, Tb.Sp*_CV_, Tb.Th*_CV_, Tb.N*_CV_, and DA_CV_), and (3) *global heterogeneity*, the standard deviation of Tb.Sp* on the entire vertebral trabecular volume (Tb.Sp*SD), reflecting the heterogeneity of the trabecular network.(13)

### Mechanical testing

Soft tissues and posterior arches were removed. Then the vertebral bodies were thawed and maintained moist at 4°C with Ashman's solution until mechanical testing.(19,20)

Before testing, a polyester resin interface (Soloplast V11, Vosschemie, Saint-Egrève, France) with a quick-setting polymer at low temperature (exothermic peak of resin polymerization ≤ 40°C) was applied to each endplate of the vertebral body to achieve parallel surfaces for load application. Then quasi-static uniaxial compressive testing was performed on the whole vertebral body submerged in Ashman's solution at 37°C with a screw-driven machine (Schenck RSA-250, Darmstadt, Germany) under displacement control (0.5 mm/s) until failure. The compressive load and displacement were assessed, respectively, by a 5000-N load cell (TME, F 501 TC, Toulon, France) and a displacement transducer mounted directly on the vertebral resin endplates (Mécanium, Lyon, France). Preconditioning was performed prior to testing (10 cycles with loading at 100 N and unloading at 50 N).

The following parameters were measured from the load-displacement data: failure load (N), defined by the force at maximum on the load-displacement curve; compressive stiffness (N/mm), defined by the linear part of the load-displacement curve slope between 25% and 75% of the failure load; and work to failure (N · mm), defined by the total area under the load-displacement curve. Because of vertebral shape, measurement of the cross-sectional area was highly variable, and therefore, estimated material properties (ie, ultimate stress and Young's modulus) were not computed.

### Statistical analysis

Shapiro-Wilk tests were used to assess whether the variables were normally distributed. Most parameters were normally distributed, except for work to failure, BV/TV_ratio_, SMI_ratio_, SMI_CV_ on the posterior biopsy, Tb.Th* and Tb.Sp* on the superior core of the anterior biopsy, Tb.Sp* on the inferior core of the anterior biopsy, Tb.Th*_CV_ and DA_CV_ on the anterior biopsy, Tb.Sp* on anterior and posterior biopsies, and Tb.Sp*SD, which were normalized using logarithmic transformation.

Data are presented as the mean ± SD. The following tests were used: (1) Mann-Whitney–Wilcoxon test for the comparison between sexes, (2) Pearson coefficients of correlation for analysis of the relationships between two variables, (3) paired *t* test for comparison between anterior and posterior virtual biopsy parameters, (4) Friedman ANOVA tests for analysis of the relationships among superior, middle, and inferior zones of the virtual biopsy and post hoc paired *t* test for vertical parameters, (5) stepwise forward multiple regression models including semipartial correlations for the selection of variables explaining mechanical testing, and 6) partial correlations with adjustments for bone mass. To adjust for multiple comparisons, the threshold for significance was fixed at a *p* value of .026 or less after the Holm-Bonferroni correction.(24) All statistical analyses were performed using SPSS 16.0 (SPSS, Inc., Chicago, IL, USA).

## Results

### Characteristics of samples and heterogeneity of vertebral trabecular bone

On the whole vertebral body, BMD averaged 0.62 ± 0.12 g/cm^2^. Mean failure load was 2615 ± 1136 N, mean stiffness was 2938 ± 1585 N/mm, and mean work to failure was 1730 ± 1129 N · mm. Descriptive statistics for trabecular microarchitectural parameters are shown in Table 1. The Kellgren-Lawrence OA score did not differ between male and female donors, and there were no significant associations between Kellgren-Lawrence grades and BMD, microarchitecture, or mechanical parameters.

**Table 1:** Trabecular Architecture Variation in the L_3_ Vertebral Body (Mean ± SD)

			Anterior core				Posterior core			
				
		Vertebral body	Total†	Superior region‡	Middle region‡	Inferior region‡	Total†	Superior region‡	Middle region‡	Inferior region‡
Trabecular microarchitecture	BV/TV (%)	13.5 (5.9)	11.4 (6.1)	**10.8 (6.9)**‡	**13.2 (7.2)**‡	**10.3 (6.1)**‡	13.5 (6.7)	**10.8 (5.7)**‡	**16.7 (8.5)**‡	**12.9 (7.6)**‡
	SMI (*n*)	2.61 (0.53)	2.75 (0.65)	**2.90 (0.66)**‡	**2.47 (0.75)**‡	**2.91 (0.69)**‡	2.76 (0.50)	**3.09 (0.41)**‡	**2.21 (0.72)**‡	**2.92 (0.55)**‡
	Tb.Sp* (µm)	1363 (332)	**1368 (382)**†	1354 (440)	1285 (327)	1301 (306)	**1209 (319)**†	**1220 (364)**‡	**1225 (353)**‡	**1055 (260)**‡
	Tb.Th* (µm)	308 (43)	288 (64)	281(75)^a^,‡	289 (59)^a^,‡	266 (47)^a^,‡	293 (42)	**272 (46)**‡	**308 (47)**‡	**270 (42)**‡
	Tb.N* (*n*/mm)	0.76 (0.16)	**0.76 (0.17)**†	0.78 (0.19)	0.81 (0.18)	0.80 (0.16)	**0.86 (0.20)**†	**0.86 (0.20)**‡	**0.88 (0.21)**‡	**0.97 (0.23)**‡
	DA (*n*)	1.46 (0.11)	**1.72 (0.17)**†	1.72 (0.19)	1.79 (0.21)	1.77 (0.21)	**1.47 (0.14)**†	1.53 (0.20)	1.40 (0.16)	1.48 (0.17)

† Anteroposterior comparison: Paired *t* tests.

‡ Vertical comparison: Friedman ANOVA tests. **Bold:** *p* ≤ .026

a 026 < *p* < .05.

There were no differences between specimens from male and female donors, except for vertical heterogeneity expressed by BV/TV_CV_, which was greater in males than in females (0.36 ± 0.17 versus 0.19 ± 0.08, *p* = .008).

Despite our limited age range, Tb.N* on the whole trabecular area and Tb.N* and Tb.Sp* on the anterior biopsy decreased significantly with age (*r* = –0.51, –0.56, and 0.55; *p* = .02, .008, and .01, respectively). No significant correlation was found between age and microarchitecture parameters from the posterior region.

Trabecular architecture was more deteriorated in the anterior versus posterior region, with lower Tb.N* (*p* = .004) and higher Tb.Sp* (*p* = .0001) and DA (*p* = .0001) in the anterior core (Table 1).

In the anterior biopsy, the three vertical regions differed significantly for BV/TV and SMI (*p* = .0001 and .021, respectively; Table 1). Using post hoc tests on these parameters, the middle region had a higher BV/TV and a lower SMI than the inferior and superior regions (*p* = .0004 to .004).

In the posterior biopsy, the three vertical regions were significantly heterogeneous for BV/TV, SMI, Tb.N*, Tb.Sp*, and Tb.Th* (*p* = .0005 to .013; Table 1), with the middle region characterized by a higher BV/TV and Tb.Th* and a lower SMI than the inferior and superior regions (*p* = .0026 to .0005). The superior and middle regions had a higher Tb.Sp* and a lower Tb.N* than the inferior region (*p* = .026 to .001).

Relationship among bone mass, trabecular microarchitecture, and vertebral mechanical behavior (Table 2).

**Table 2:** Pearson's Correlation Coefficients (*r*) Among Bone Mass, Trabecular Microarchitecture, and Biomechanical Properties of the Vertebral Body

	Failure load	Work to failure	Stiffness
BMD	**0.66**	**0.58**	**0.54**
BV/TV	**0.73**	0.43	**0.66**
SMI	**−0.81**	−0.45^a^	**−0.66**
Tb.Sp*	**−0.57**	−0.30	**0.62**
Tb.Th*	0.44^a^	0.39	0.30
Tb.N*	**0.51**	0.23	**0.58**
DA	0.38	0.10	0.29
Tb.Sp*SD	−0.36	−0.13	**−0.49**

*Note:* Abbreviations defined in the methods section.

**Bold:** *p* ≤ .026

a 026 < *p* < .05.

#### Bone mass

BMD and BV/TV were significantly positively correlated with failure load, work to failure, and stiffness (*r* = 0.54 to 0.73; *p* = .01 to <.0001; Table 2).

#### Trabecular microarchitecture

BV/TV, SMI, Tb.N*, and Tb.Sp* were significantly correlated with failure load and stiffness (|*r*| = 0.51 to 0.81; *p* = .019 to <.0001; Table 2) but were not related to work to failure.

In multiple regression models using the following equation: mechanical behavior = bone mass + microarchitecture, with mechanical behavior corresponding to failure load or stiffness or work to failure, bone mass corresponding to BMD or BV/TV, and trabecular microarchitectural parameters corresponding to SMI, DA, Tb.Sp*, and Tb.Th*, SMI appeared to be the most pertinent parameter to predict mechanical behavior because it was always the first to be included in the stepwise regression analysis.

Relationship between trabecular microarchitecture heterogeneity and vertebral body mechanical behavior.

#### Global heterogeneity

Tb.Sp*SD of the entire vertebral trabecular bone region was negatively correlated with stiffness (*r* = –0.49; *p* = .023).

#### Anteroposterior heterogeneity

For the anterior biopsy, all trabecular microarchitectural parameters except Tb.Th* and DA were correlated with failure load and stiffness (|*r*| = 0.50 to 0.74; *p* = .001 to .0001; Table 3). For the posterior biopsy, only BV/TV was correlated with failure load and BV/TV and SMI with stiffness. None of the architecture parameters from the anterior and posterior cores were significantly correlated with work to failure.

**Table 3:** Pearson's Correlation Coefficients Between Trabecular Microarchitecture and Anteroposterior Heterogeneity (Ratios) With Mechanical Parameters

		Failure load	Work to failure	Stiffness
Anterior biopsy microarchitecture	BV/TV	**0.67**	0.47^a^	**0.50**
	SMI	**−0.74**	−0.46^a^	**−0.55**
	Tb.Sp*	**−0.61**	−0.35	**−0.59**
	Tb.Th*	0.29	0.34	0.09
	Tb.N*	**0.56**	0.30	**0.57**
	DA	0.38	0.17	0.31
Posterior biopsy microarchitecture	BV/TV	0.47^a^	0.15	**0.51**
	SMI	**−0.61**	−0.27	**−0.52**
	Tb.Sp*	−0.35	−0.04	−0.46^a^
	Tb.Th*	0.42	0.26	0.39
	Tb.N*	0.27	0.01	0.39
	DA	0.25	0.14	0.03
Anteroposterior heterogeneity	BV/TV_ratio_	**−0.53**	**−0.57**	−0.24
	SMI_ratio_	0.31	0.29	0.16
	Tb.Sp*_ratio_	0.32	0.39	0.14
	Tb.Th*_ratio_	−0.13	−0.36	0.19
	Tb.N*_ratio_	−0.36	−0.39	−0.21
	DA_ratio_	−0.10	−0.01	−0.25

**Bold:** *p* ≤ .026

a 026 < *p* < .05.

In multiple regression models using the following equation: mechanical behavior = anterior microarchitecture + posterior microarchitecture, with mechanical behavior corresponding to failure load or stiffness or work to failure and microarchitecture corresponding to SMI, DA, Tb.Sp*, and Tb.Th*, the posterior parameter was consistently excluded from the model. As a result, parameters of the anterior biopsy were the best predictors of mechanical behavior.

Considering the heterogeneity parameters, BV/TV_ratio_ was significantly negatively correlated with failure load and work to failure (*r* = –0.53 and –0.57; *p* = .013 and .007, respectively; Table 3). No other anteroposterior ratios were correlated with vertebral mechanical properties.

#### Vertical heterogeneity

Since trabecular microarchitectural parameters of the anterior biopsy were the best predictors of mechanical behavior, we studied vertical heterogeneity only on the anterior biopsy.

In the superior region, BV/TV and SMI were significantly correlated with failure load and work to failure (|*r*| = 0.49 to 0.65; *p* = .025 to .001); Tb.Sp* also was correlated with failure load (*r* = –0.61; *p* = .003). Only Tb.Sp* and Tb.N* were significantly correlated with stiffness (*r* = –0.50 and 0.49, respectively; *p* = .02). In the middle region, BV/TV, SMI, and Tb.Sp* were significantly correlated with failure load and stiffness (|*r*| = 0.48 to 0.68; *p* = .026 to .001). In the inferior region, all trabecular microarchitectural parameters were significantly correlated with failure load and stiffness (|*r*| = 0.52 to 0.71; *p* = .015 to .0001) except that DA was not related to failure load and stiffness, and Tb.Th* was not related to stiffness (Table 4). No significant correlations were found with work to failure.

**Table 4:** Pearson's Correlation Coefficients Among Trabecular Microarchitecture, Vertical Heterogeneity (CV), and Vertebral Mechanical Properties in the Three Vertical Regions of the Anterior Biopsy

		Failure load	Work to failure	Stiffness
Superior region	BV/TV	**0.49**	**0.57**	0.19
	SMI	**−0.63**	**−0.65**	−0.26
	Tb.Sp*	**−0.61**	−0.46^a^	**−0.50**
	Tb.Th*	0.29	0.47^a^	0.01
	Tb.N*	**0.57**	0.41	**0.49**
	DA	0.41	0.32	0.15
Middle region	BV/TV	**0.62**	0.34	**0.50**^a^
	SMI	**−0.68**	−0.40	**−0.52**
	Tb.Sp*	**−0.51**	−0.28	**−0.48**
	Tb.Th*	0.36	0.24	0.23
	Tb.N*	0.42	0.17	0.43
	DA	0.24	0.06	0.23
Inferior region	BV/TV	**0.71**	0.32	**0.67**
	SMI	**−0.66**	−0.19	**−0.63**
	Tb.Sp*	**−0.54**	−0.22	**−0.62**
	Tb.Th*	**0.59**	0.38	0.44^a^
	Tb.N*	**0.53**	0.21	**0.63**
	DA	0.13	−0.08	0.13
Vertical heterogeneity	BV/TV_CV_	−0.29	−0.03	−0.32
	SMI_CV_	0.46^a^	0.35	0.30
	Tb.Sp*_CV_	−0.20	−0.25	−0.17
	Tb.Th*_CV_	−0.13	0.00	−0.22
	Tb.N*_CV_	−0.16	−0.26	−0.13
	DA_CV_	0.13	0.26	−0.20

**Bold:** *p* ≤ .026

a 026 < *p* < .05.

Regarding the vertical heterogeneity parameters (BV/TV_CV_, SMI_CV_, Tb.Sp*_CV_, Tb.Th*_CV_, Tb.N*_CV_, and DA_CV_), none were significantly correlated with mechanical behavior.

Relative role of bone mass parameters, trabecular microarchitecture, and its heterogeneity parameters on mechanical behavior.

To determine the relative contribution of heterogeneity parameters to vertebral mechanical behavior, we performed multiple regression models using the following equation: mechanical behavior = bone mass + microarchitecture + microarchitectural heterogeneity, with mechanical behavior corresponding to failure load or stiffness or work to failure, bone mass corresponding to BMD or BV/TV, microarchitecture as the most pertinent parameter corresponding to SMI, and heterogeneity parameters corresponding to all anteroposterior ratios, vertical CV, and Tb.Sp*SD. For mechanical behavior and heterogeneity parameters, only failure load and DA_ratio_ presented with a significant introduction in the equations.

The combination of BMD (third step, *p* = .004), SMI (first step, *p* < .0001), and DA_ratio_ (second step, *p* = .001) was significant for failure load (*r* = 0.93; *p* < .0001). Also, the combination of BV/TV (*p* = n.s.), SMI (second step, *p* = .008), and DA_ratio_ (first step, *p* = .003) was correlated with failure load (*r* = 0.89; *p* < .0001; Table 5).

**Table 5:** Multiple Regression Analysis Including the Coefficient of Determination (*R*^2^), the *p* Value, and Semipartial Correlation (*r*^2^) for Each Variable Included in the Models

Variables				
			
Dependent	Independent	Final *R*^2^	Semipartial correlation (*r*^2^)	*p* Value
Failure load				
	BMD		0.10	.004
	SMI		0.39	<.0001
	DA_ratio_		0.14	.001
		0.86		<.0001
Failure load				
	BV/TV		0.03	n.s.
	SMI		0.11	.008
	DA_ratio_		0.14	.003
		0.80		<.0001

Furthermore, the correlation between failure load and DA_ratio_ remained significant after adjustment with bone mass (ie, BV/TV; *r* = 0.57; *p* = .009).

## Discussion

The aim of this study was to determine the contribution of trabecular microarchitecture and its heterogeneity to the mechanical behavior of human lumbar vertebrae. We assessed trabecular microarchitectural heterogeneity parameters in several ways: (1) by the ratio of anteroposterior trabecular microarchitecture values, (2) by the coefficient of variation of trabecular microarchitecture values in superior, middle, and inferior regions, and (3) by the standard deviation of trabecular separation across the entire vertebral trabecular volume.

Consistent with previous studies, we observed marked heterogeneity of vertebral trabecular architecture, with the anterior region showing impaired trabecular architecture compared with the posterior region.(8–12) Correlations between mechanical behavior and microarchitecture varied within vertebral regions but generally indicated that the anterior part of the lumbar vertebral body is more strongly related to vertebral mechanical properties and therefore may be a better region to measure when predicting vertebral fracture risk. We also found that in this sample of vertebrae from middle- to old-aged donors, trabecular alterations were characterized not only by a reduction in bone mass but also by changes in microarchitecture that taken together improve prediction of vertebral mechanical properties.(6,7,12) Specifically, BMD alone explained up to 44% of the variability of the mechanical behavior; BV/TV alone, up to 53%; and SMI alone, up to 66%. However, bone mass parameters (ie, BMD or BV/TV) in combination with trabecular microarchitecture (ie, SMI) and its heterogeneity (ie, DA_ratio_) improved the prediction of vertebral mechanical behavior markedly, together explaining up to 86% of the variability in biomechanical properties.

Vertebral trabecular bone has a 3D microarchitecture that consists of interconnecting plates and rods. The plate versus rod nature of the vertebral trabecular bone can be determined using the structure model index (SMI), which has been shown previously to be correlated with mechanical properties of trabecular bone.(25–27) Moreover, in young individuals, there are twice as many vertical trabeculae than horizontal ones, and this ratio of vertical to horizontal trabeculae increases with age.(26) Along with this relatively greater loss of horizontal trabecular is thinning of horizontal trabeculae, whereas the remaining vertical trabeculae tend to maintain their thickness with advancing age and even may increase in thickness.(25,26) In such a structure, the degree of anisotropy (DA) reflects the preferential vertical alignment of trabeculae. Thus, as bone loss progresses, the deterioration of the vertebral trabecular architecture results in a more anisotropic structure with a greater susceptibility to fracture. Interestingly, in our study, the global DA was not correlated with vertebral mechanical behavior; however, the anteroposterior heterogeneity of DA (DA_ratio_) was. This role of anisotropic heterogeneity appeared when the DA_ratio_ was included in multiple regression analyses in combination with bone mass parameters and SMI. The significance of the DA_ratio_ may be explained in part by our elderly donors, who have very low BMD and BV/TV values, perhaps providing a greater opportunity for the DA_ratio_ to influence mechanical behavior. Altogether these findings suggest that anteroposterior variation of trabecular alignment explained mechanical behavior better than DA measured in the entire trabecular region, highlighting the potential usefulness of DA_ratio_ for predicting vertebral mechanical behavior.

In a previous study of the femoral neck using micro–computed tomography (µCT), Ciarelli and colleagues showed that patients with hip fracture had a significantly more anisotropic structure than those in a control group after adjustment for bone mass.(28) Similar to conclusions of this study, we suggest that, for a population with similar bone volume fraction, the likelihood for fracture may be influenced by the heterogeneity of anisotropy in the trabecular bone structure.

In addition, our finding that Tb.Sp*SD is negatively correlated with vertebral mechanical properties is consistent with clinical studies that have measured Tb.Sp*SD at peripheral skeletal sites and reported higher values in women with a history of fragility fracture.(13–17)

Our study had several limitations. First, trabecular bone structure was measured using an 82-µm isotropic voxel size, which may have led to an overestimation of some microarchitectural features.(29,30) Because of partial-volume effects with lower-resolution images, BV/TV and Tb.Th can be overestimated and Tb.Sp underestimated when compared with “gold standard” µCT or histomorphometry.(31,32) However, several studies have compared microarchitectural measurements made with an 82-µm voxel size and greater with those obtained with µCT and found very high correlations between the microarchitectural parameters.(32,33) Second, we recognize that images of this high resolution are not currently used clinically in the axial skeleton. However, recent studies have shown that microarchitectural measurements acquired using high-resolution multidetector CT (MDCT) imaging available in vivo correlate strongly with those assessed using either µCT or HR-pQCT.(34,35) Accordingly, MDCT is quite promising for assessment of trabecular and cortical microarchitecture in the spine and assessment of microarchitecture and its heterogeneity as performed in our study. Indeed, our results provide a strong rationale to conduct a clinical study testing whether heterogeneity measures improve identification of patients at risk for vertebral fracture. Third, the loading mode used was uniaxial compression. Because most osteoporotic vertebral fractures are anterior wedge fractures, the response to combined compression and anteroposterior bending also may be of interest.(36) It is possible that in this “physiologic” mechanical condition of compression and anteroposterior bending, BMD would be an even worse predictor of vertebral mechanical behavior with a greater contribution of trabecular microarchitecture and its heterogeneity, particularly at the anterior region. This could be assessed in further experimental studies and in those that use finite-element analysis (FEA) models to simulate different loading modes. Another limitation of our study is the inability to know how loads are distributed between cortical and trabecular bone in the tested loading conditions as well as loading conditions seen in vivo. Obviously, FEA could provide some of this information and could extend the current experimental observations. Finally, this study did not take in account other factors such as bone tissue composition (ie, degree of mineralization, collagen maturity and cross-link characteristics, and crystal size and perfection) or cortical shell morphology, which also may contribute to vertebral strength.(37–41)

In conclusion, our data indicate that assessment of trabecular microarchitecture and its regional heterogeneity may enhance prediction of vertebral fracture risk, and accordingly, therapies that maintain microarchitecture and reduce heterogeneity would preserve vertebral strength to a greater extent than changes in BMD alone.
